# A Multifaceted Approach to Seizure Management in a Patient With Down Syndrome, Obstructive Sleep Apnea, and Hypothyroidism: A Case Report

**DOI:** 10.7759/cureus.55465

**Published:** 2024-03-03

**Authors:** Takafumi Uchi, Shingo Konno, Hideo Kihara, Hideki Sugimoto

**Affiliations:** 1 Department of Neurology, Toho University Ohashi Medical Center, Tokyo, JPN

**Keywords:** anti-seizure medication, continuous positive airway pressure, obstructive sleep apnea, seizure, down syndrome

## Abstract

In this case study, a 16-year-old male with Down syndrome (DS) faced persistent nocturnal seizures despite anti-seizure medications and treatment for concurrent hypothyroidism. Obstructive sleep apnea (OSA), a common issue in patients with Down syndrome, was revealed as a trigger of the seizures. The implementation of continuous positive airway pressure (CPAP) therapy along with medication adjustments led to a significant decrease in seizure frequency, highlighting the importance of a comprehensive approach to seizure management in patients with complex medical conditions.

## Introduction

The prevalence of epilepsy in individuals with Down syndrome (DS) is higher than that in the general population, ranging from 1% to 13% [1,2]. A study of 85 patients with DS in three age groups (14-16 years, 23-29 years, and 50-60 years) found that the prevalence of epilepsy increased with age [3]. Forty percent of individuals develop seizures before one year of age, and another 40% develop seizures in their 30s or later. Males have an earlier age of onset [1]. The types of seizures in individuals with DS are 47% partial seizures, 32% infantile spasms, and 21% generalized tonic-clonic seizures [1]. The mechanisms underlying the high seizure susceptibility in DS have not been thoroughly explained; however, seizures in infancy may be due to inherent structural brain abnormalities [1]. The reported prevalence of epilepsy in adolescents and young adults with DS varies from 8% to 14% [2]. Data on epilepsy prevalence rates in individuals with DS vary owing to differences in study populations, observation periods, and other methodological issues [2].

The prevalence of obstructive sleep apnea (OSA) in children with DS is reported to be between 30% and 76% [4,5]. A meta-analytic literature review of 23 studies examining OSA among 1,469 people with DS found a pooled prevalence of OSA of 69.6% in the referred patients [6]. Additionally, a survey by Maris et al. found that 66% of a large group of unselected patients with DS had OSA on full-night polysomnography [7]. Another study reported the prevalence of OSA in children with DS ranging from 69% to 76% based on different criteria for defining OSA [8]. Factors for OSA in individuals with DS include midfacial hypoplasia, mandibular hypoplasia, glossoptosis, an abnormally small upper airway with superficially positioned tonsils, and relative tonsillar and adenoidal encroachment [5].

The treatment of OSA in epileptic patients without DS can lead to several potential positive outcomes. Existing literature suggests that OSA treatment can improve seizure control in patients with epilepsy. The mechanisms through which OSA treatment may suppress epilepsy include improvement of seizure control, reduction of seizure frequency, and higher seizure-free rate [9]. The proposed mechanisms underlying the positive effects of OSA treatment on epilepsy include ameliorating factors such as sleep deprivation, sleep fragmentation, cerebral hypoxemia, decreased cardiac output, cardiac arrhythmias, autonomic instability, and increased sympathetic activity [9].

Here, we report the management of seizures using a multifaceted approach in a patient with Down syndrome and OSA.

## Case presentation

**Figure 1:**
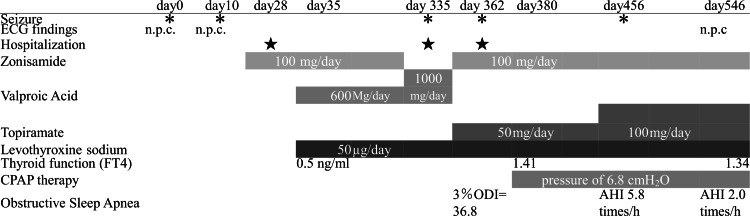
Timeline of events This figure shows the timeline of clinical events and treatment adjustments for our patient with seizures and OSA. It includes the dates of hospitalization, seizure occurrence, medication dosages (zonisamide, valproic acid, topiramate, and levothyroxine sodium), thyroid function tests (FT4 ng/mL), and continuous positive airway pressure therapy details. Significant clinical changes are marked with symbols, and the timeline spans from days 0-546, highlighting the changes in treatment and clinical outcomes over time. AHI: Apnea-Hypopnea Index, CPAP: continuous positive airway pressure, ECG: electrocardiogram, n.p.c.: no paroxysmal change, ODI: Oxygen Desaturation Index, OSA: obstructive sleep apnea

The patient's clinical course is presented in Figure 1.

A 16-year-old male with trisomy 21 was born without complications during pregnancy or birth. He had a slightly lower birth weight and achieved milestones such as sitting, crawling, and walking several months later than normal children. Growth rates continued to slow during childhood and were shorter than his peers. However, the weight gradually increased from the school-age period. Mild intellectual disabilities affected learning, but he received support and education tailored to his abilities and needs. Language development was also delayed. However, he had not experienced a loss of consciousness or febrile convulsions until the first admission.

**Figure 2:**
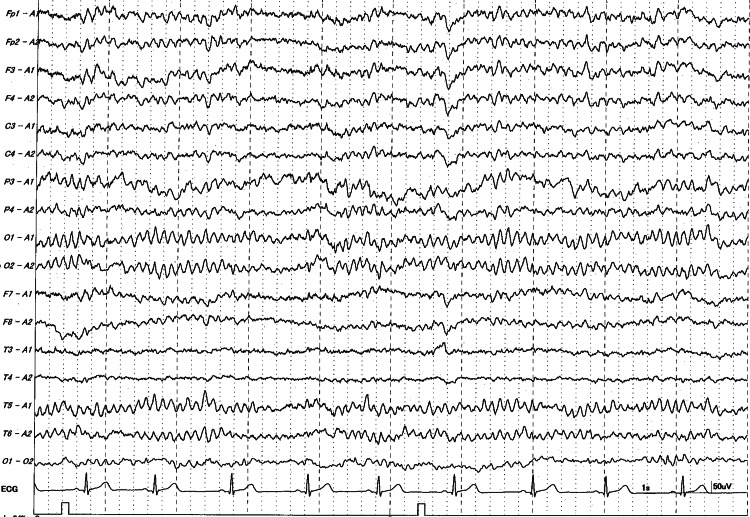
The first electroencephalograph of the patient Electroencephalography showed typical background rhythms with reactive 10 Hz alpha waves and occasional theta waves in the frontal, central, and parietal leads while awake. In addition, the responses to photic stimulation and hyperventilation were normal.

He was placed in our care experiencing tonic seizures in the early morning. His whole body had suddenly stiffened without previous symptoms, and fine spasms had continued symmetrically for approximately 10-20 seconds. His hands and feet had been firmly thrust, and his body had been thrown back. The fine spasms had gradually become rhythmic movements that lasted for 30-60 seconds before subsiding. After a few minutes, the convulsions had subsided; however, the patient had bitten his tongue. He had slowly regained consciousness in a post-seizure state. He visited the emergency department of our hospital (day 0). Initial electroencephalography (EEG) revealed no epileptiform discharges (Figure 2).

**Figure 3:**
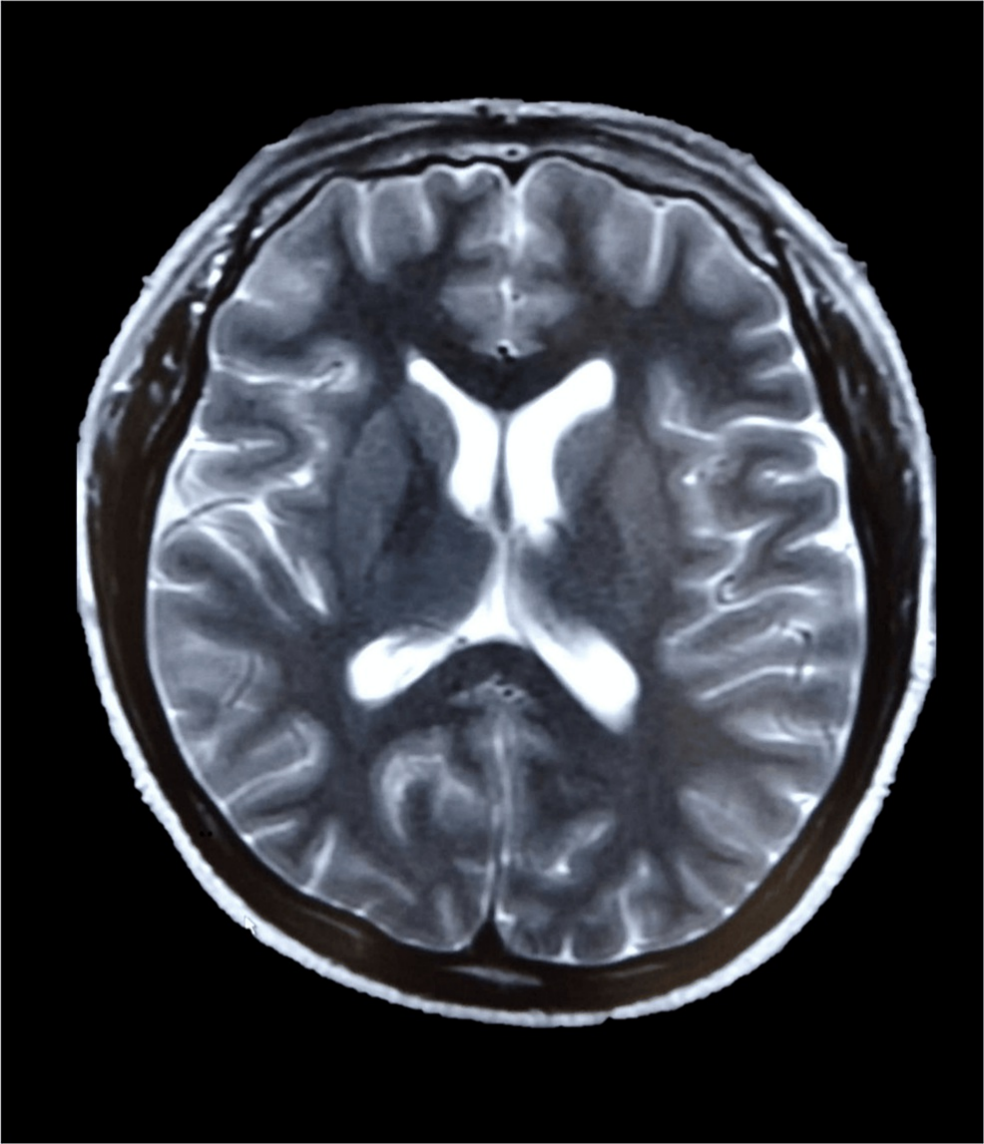
Brain magnetic resonance imaging of the patient on admission This picture illustrates a simple T2-weighted image of the brain magnetic resonance imaging. No abnormalities are identified. Normal brain tissue appears uniformly represented. The brain parenchyma, ventricles, and cerebrospinal fluid are clearly visualized, with no abnormal findings.

The brain magnetic resonance imaging (MRI) did not show abnormal findings (Figure 3), leading us to monitor him clinically without intervention.

The patient subsequently experienced a second seizure episode, necessitating admission to an urgent care facility (day 10). On the first admission, his consciousness recovered. He was afebrile, was 158 cm tall, weighed 72 kg (body mass index of 28.8), and had no abnormalities in his chest and abdomen. Pitting edema was observed in the lower extremities. Although a re-examination of the EEG did not reveal paroxysmal changes, we initiated treatment with zonisamide (100 mg, daily), which has a low effect on weight gain (day 28). The laboratory data showed high thyroid-stimulating hormone (TSH) levels at 91.1 µIU/mL (normal: 0.2-3.2 µIU/mL), low free triiodothyronine (FT3) levels at 0.51 ng/dL (normal: 2.9-6 ng/dL), and low free thyroxine (FT4) levels at 0.51 ng/dL (normal: 0.78-2.10 ng/dL). The anti-thyroglobulin and anti-thyroid peroxidase antibody levels were 64.5 IU/mL (normal: <19.3 IU/mL) and 2,330 IU/mL (normal: <3.3 IU/mL), respectively, and ultrasound echo examination showed swelling of the thyroid isthmus. He was therefore diagnosed with Hashimoto's disease and started receiving levothyroxine sodium at 50 μg/day (day 35).

**Figure 4:**
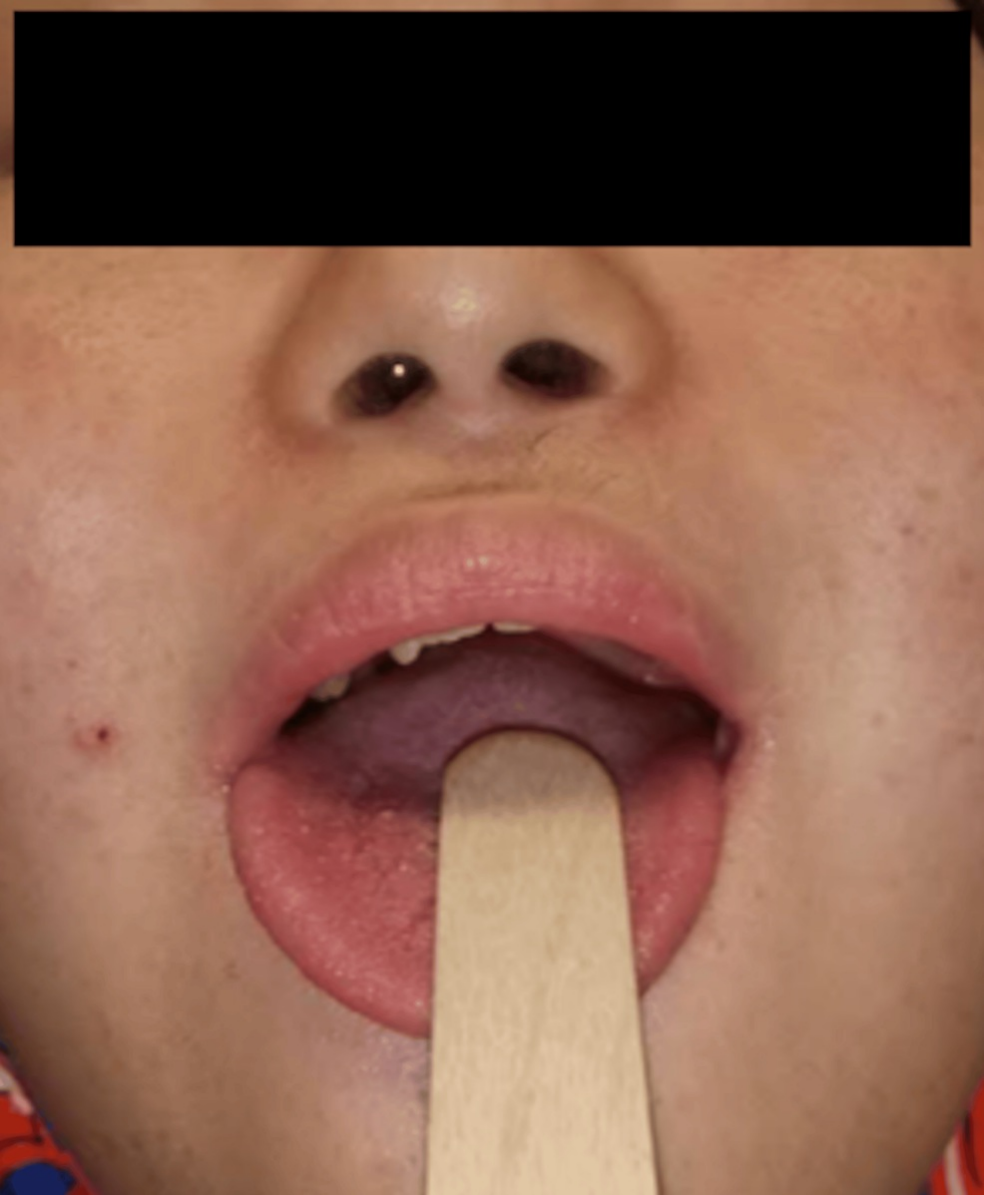
Image of the patient's oral cavity The image shows a close-up view of the patient with an open mouth, with the tongue protruding as far as possible. Although a wooden depressor was used to gently lower the tongue to obtain a clear view of the mouth, we only observed his soft palate. The modified Mallampati classification, which has practical value in clinical settings and prospective studies on sleep-disordered breathing, was grade IV, where only the hard palate was visible.

Ten months later, the patient experienced another seizure, which led us to change his medication to valproic acid (VPA), beginning at 600 mg/day. His initial VPA level was 50 µg/mL, which was within the therapeutic range of 50-100 µg/mL but indicated a need to adjust the dose (day 335). Following another seizure within a month, we increased his VPA to 1,000 mg/day, achieving a serum level of 80 µg/mL. Despite treatment with levothyroxine sodium, his hydroid function recovered normally. Fifteen days after being discharged from the hospital, he had another seizure requiring readmission (day 362). At this point, thyroid function was normal: TSH level was 0.84 ng/dL, FT3 level was 2.9 pg/mL, and FT4 level was 1.41 ng/dL. We added topiramate (50 mg/day) to the VPA because of the drowsiness caused by VPA at 1,000 mg/day. Notably, his seizures were mainly occurring at night or in the early morning. A type III portable monitor test revealed that the Respiratory Event Index and 3% Oxygen Desaturation Index were 36.8 (severe: >30) and 45.6, respectively. These results confirmed severe OSA owing to obesity, a short neck, macroglossia associated with DS (Figure 4), and neck soft tissue edema from hypothyroidism, which had possibly triggered the seizures.

Continuous positive airway pressure (CPAP) therapy (Sleep mate® 10 Auto, TEIJIN Medical, Osaka, Japan) was introduced in auto mode with a median pressure of 6.8 cmH20. The patient's OSA condition improved, with an Apnea-Hypopnea Index (AHI) of 5.8 times/hour and a markedly reduced frequency of seizure episodes (day 380). However, despite the CPAP therapy, the patient experienced seizures on two occasions within six months (day 456). Increasing the topiramate dose to 100 mg/day effectively controlled subsequent seizures. Three months later (day 546), a re-examination of the EEG did not reveal any paroxysmal changes, and AHI was 2 times/hour.

## Discussion

Two key outcomes were observed. We discovered that nocturnal seizures in adolescents with DS were closely associated with OSA. This relationship is supported by existing research that shows a higher incidence of epilepsy in individuals with DS, particularly after the age of 40 years [2], and a well-documented link between DS and sleep disorders such as OSA [5]. These sleep disorders may cause nighttime disturbances, including trouble falling asleep, fragmented sleep, and seizures triggered by reduced oxygen levels.

Interestingly, while CPAP therapy proved effective in reducing these seizures, the continued need for anti-seizure medication suggests a more profound organic brain disorder, possibly due to long-term hypoxia. This aligns with the understanding that OSA may induce seizures through sleep disruption, sleep deprivation, and cerebral hypoxemia, leading to oxidative stress [10].

Contrary to our findings, some studies have not shown a direct link between OSA and increased seizure frequency in individuals with DS, suggesting the presence of other potential factors in seizure manifestation [11]. Nonetheless, the high prevalence of undetected sleep disorders in adults with DS emphasizes the importance of comprehensive sleep assessment [12]. Alternatives to CPAP therapy, such as hypoglossal nerve stimulation, have been suggested and are well-tolerated in adolescents with DS [13], indicating the need to explore various treatment options.

Our findings contribute significantly to our understanding of the clinical implications of sleep-induced seizures in adolescents with DS [2,14]. They highlight the necessity of regular screening for OSA as part of a neurological assessment and suggest an integrated treatment approach that addresses both the respiratory and neurological aspects of DS. The effectiveness of CPAP therapy in reducing seizure frequency despite the need for anti-seizure medication indicates the complexity of the underlying neurological issues, likely exacerbated by chronic hypoxia [15].

However, this study had some limitations. Because it is based on a single case, its generalizability is limited. Further studies with a larger sample size and controlled experimental design could provide more substantial evidence. Additionally, the impact of factors such as medication changes or lifestyle modifications on seizure frequency must be fully explored. Despite these limitations, our study underscores the significant interplay between sleep disorders and the neurological manifestations of DS, offering valuable insights for clinicians and researchers.

Furthermore, unfortunately, idiopathic epilepsy could not be completely ruled out because continuous EEG could not be performed. However, his seizures always occurred during sleep or early in the morning, suggesting that hypoxia during sleep may have triggered them. This report describes only one case, and the substantiation of our findings will depend on the accumulation of future cases.

## Conclusions

In conclusion, in patients with DS, where the frequency of epilepsy is notably higher than that in the general population, the presence of sleep apnea could further complicate the clinical picture. While it is not possible to rule out epilepsy based solely on the presence of sleep apnea, understanding and addressing the interplay between these conditions is crucial for effective management and treatment.
